# Metabolic Drivers of Valve Calcification and Atrial Remodeling in Calcific Aortic Stenosis

**DOI:** 10.3390/biom16020251

**Published:** 2026-02-04

**Authors:** Simina Mariana Moroz, Alina Gabriela Negru, Silvia Luca, Mihaela Valcovici, Mirela Baba, Alina Maria Lupu, Ana Lascu, Daniel Florin Lighezan, Ioana Mozos

**Affiliations:** 1 Center for Advanced Research in Cardiovascular Pathology and Hemostaseology, Victor Babeş University of Medicine and Pharmacy, 300041 Timișoara, Romania; simina.moroz@umft.ro (S.M.M.);; 2 Doctoral School Medicine-Pharmacy, Victor Babeş University of Medicine and Pharmacy, 300041 Timișoara, Romania; mirela.baba@umft.ro; 3 Cardiology Department, Victor Babeş University of Medicine and Pharmacy, 300041 Timișoara, Romania; 4 Institute of Cardiovascular Diseases Timișoara, 300310 Timișoara, Romania; 5 Research Center of the Institute of Cardiovascular Diseases Timișoara, 300310 Timișoara, Romania; 6 Center for Translational Research and Systems Medicine, Victor Babeş University of Medicine and Pharmacy, 300041 Timișoara, Romania; 7 Center for Diagnosis and Study of Parasitic Diseases, Victor Babeş University of Medicine and Pharmacy, 300041 Timișoara, Romania; lupu.alina@umft.ro; 8 Clinical Laboratory, Institute of Cardiovascular Diseases Timișoara, 300310 Timișoara, Romania; 9 Department of Functional Sciences-Pathophysiology, Victor Babeş University of Medicine and Pharmacy, 300041 Timișoara, Romania; 10 Department of Internal Medicine I-Medical Semiotics I, Victor Babeş University of Medicine and Pharmacy, 300041 Timișoara, Romania

**Keywords:** aortic stenosis, metabolomics, valve calcification, atrial remodeling, mitochondrial dysfunction, gut microbiome, transcatheter aortic valve replacement

## Abstract

AS, one of the most common forms of valvular heart disease, requiring intervention in aging populations in Europe and North America, has traditionally been viewed as a passive, degenerative condition. However, growing evidence supports a paradigm shift toward recognizing AS as an active metabolic and inflammatory disorder. This narrative review synthesizes experimental, translational, and clinical evidence published between 2015 and 2025 examining metabolic mechanisms linking valvular calcification and atrial remodeling in AS and discusses their clinical relevance in the context of transcatheter aortic valve replacement (TAVR). We discussed classical pathways involving mineral metabolism and vitamin signaling, alongside emerging roles of lipid oxidation, mitochondrial dysfunction, epigenetic regulation, and gut microbiome-derived metabolites. Further, metabolomic signatures associated with disease severity and post-TAVR outcomes were reviewed, highlighting the predominantly associative nature and current limitations of these data. Although valve replacement remains the only effective therapy for advanced AS, metabolic and multi-omics insights may improve future risk stratification and mechanistic understanding. Metabolomic profiling could be integrated at multiple points in the clinical pathway for aortic stenosis and TAVR—most promisingly for pre-procedural risk stratification. The present paper focuses on an integrative framework in which valvular calcification and atrial remodeling are viewed within a broader context of metabolic dysregulation. Future research should aim to translate molecular biomarkers into real-world diagnostics and targeted interventions.

## 1. Introduction

Aortic stenosis (AS) affects a significant proportion of the elderly population, particularly individuals over 75 years [1,2,3]. Once dismissed as a result of passive leaflet degeneration, the pathophysiology of AS is now considered an intricate process orchestrated by immune activation, oxidative injury, lipid infiltration, and metabolic dysregulation [1,4,5]. Clinically, AS is associated not only with valvular obstruction but also with profound structural and electrical remodeling of the left ventricle and atria—manifesting as hypertrophy, fibrosis, diastolic dysfunction, and atrial fibrillation (AF) [2,3,6,7]. These secondary changes critically influence prognosis following surgical or transcatheter valve interventions [6,7,8]. The disease frequently progresses to left ventricular hypertrophy, diastolic dysfunction, atrial enlargement, and eventually heart failure if left untreated [1,2,4]. With increasing life expectancy and improved management of coronary artery disease, the burden of AS is rising globally [1,2,4]. AS is now recognized as metabolically and epigenetically driven [1,4,9]. The transition from asymptomatic to symptomatic AS involves a cascade of molecular events, including valvular interstitial cell (VIC) activation, inflammation, oxidative stress, and osteogenic reprogramming. These mechanisms are influenced by lipid infiltration, vitamin K deficiency, and gut-derived toxins like Trimethylamine N-oxide (TMAO) [10,11,12,13,14]. In parallel, left atrial remodeling, characterized by fibrosis, mitochondrial dysfunction, and atrial fibrillation, emerges as a significant determinant of prognosis after TAVR [6,7,15,16]. It predisposes to atrial fibrillation and is strongly associated with adverse outcomes after TAVR, including heart failure hospitalization and reduced quality of life [6,7,15,16,17]. While growing experimental and observational data involve metabolic pathways in both valvular calcification and atrial remodeling, much of the current evidence remains associative, and direct causal relationships and therapeutic implications require prospective clinical validation.

Recent advances in systems biology and multi-omics technologies have reframed AS as a disease of metabolic disarray. High-resolution mass spectrometry and nuclear magnetic resonance (NMR)–based metabolomics have uncovered consistent metabolic signatures in AS patients, including disruptions in amino acid metabolism, impaired fatty acid oxidation, and elevated pro-inflammatory lipid mediators [15,16,18,19,20,21]. These signatures have been associated with disease progression and post-TAVR outcomes [15,16,19,20,21].

Despite these mechanistic insights, current therapy remains limited to surgical or transcatheter valve replacement, with no approved pharmacological agents capable of halting or reversing disease progression [1,2,3,4]. This therapeutic gap underscores the need for metabolic, anti-inflammatory, and molecularly targeted strategies [2,3,4,22].

Traditionally considered a result of passive wear and tear, AS is now recognized as a dynamic, cell-mediated process involving inflammatory signaling, oxidative stress, endothelial dysfunction, and osteogenic reprogramming of valvular interstitial cells (VICs) [1,4,23,24]. Moreover, left atrial and ventricular remodeling contribute to clinical symptoms and adverse post-TAVR outcomes [6,7,15]. Recently, attention has focused on metabolic contributors—ranging from nutrient deficiencies to microbiota-derived metabolites—that influence calcification, fibrosis, and arrhythmogenesis [12,13,25,26]. Moreover, the gut–heart axis has emerged as a novel modulator of both valvular and atrial remodeling. In broader cardiovascular and experimental models, microbial metabolites such as TMAO and indoxyl sulfate exacerbate inflammation and fibrogenesis via NOD-Like Receptor Pyrin Domain–Containing Protein 3 (NLRP3) inflammasome and Mitogen-Activated Protein Kinase (MAPK) pathways. At the same time, short-chain fatty acids (SCFAs), such as butyrate, exert protective roles through anti-inflammatory and epigenetic regulation [12,13,25,26].

This modern understanding points toward novel therapeutic targets, many of which lie within metabolic pathways. As such, the integration of metabolomic and systems biology approaches is reshaping how we investigate, diagnose, and potentially treat this condition [15,16,19,20,22]. By integrating metabolic drivers with clinical outcomes, we aim to provide a conceptual framework for understanding disease heterogeneity and identifying future research directions and the next generation of personalized, mechanism-based interventions in aortic stenosis.

This article aims to review experimental, translational, and clinical evidence published between 2015 and 2025, with a focus on the metabolic mechanisms linking valvular calcification and atrial remodeling in aortic stenosis. The literature was selected to reflect contemporary mechanistic and translational advances, particularly the emergence of metabolomics, epigenetics, and systems biology approaches after 2015. This article is a narrative review intended to synthesize experimental, translational, and clinical literature relevant to metabolic mechanisms in AS. The review prioritizes mechanistic plausibility and clinical relevance over former systematic selection, and most of the available evidence remains associative rather than causal.

## 2. Pathophysiology of Calcific Aortic Stenosis

Calcific aortic stenosis is an active, regulated process initiated by endothelial injury and driven by lipid infiltration, chronic inflammation, and phenotypic transformation of valvular interstitial cells (VICs) into myofibroblastic and osteoblast-like cells that deposit extracellular matrix and mineralized nodules; mechanical stress and disturbed flow further concentrate these processes on the leaflet cusps [1,4,5,23,24]. Key molecular pathways implicated include osteogenic signaling cascades (e.g., Bone Morphogenetic Protein-2 (BMP2), Runt-Related Transcription Factor 2 (RUNX2)), pro-inflammatory cytokines, and extracellular matrix remodeling enzymes that together promote progressive leaflet stiffening and obstruction [4,5,23,24].

Emerging metabolomic profiling studies revealed broader systemic and tissue-level metabolic perturbations that may link valve calcification to atrial remodeling and to variable recovery after transcatheter aortic valve replacement (TAVR), suggesting new biomarker and therapeutic pathways to explore [15,16,19,20,21,27]. Based on these mechanistic insights, a conceptual reframing of calcific aortic stenosis is proposed, as summarized in Table 1.

## 3. Metabolic Drivers of Valvular Calcification

Classical metabolic disturbances, such as dysregulated calcium-phosphate balance, impaired vitamin D signaling, and secondary hyperparathyroidism, are central contributors to valvular calcification [4,28,29,30]. Elevated circulating phosphate and calcium promote hydroxyapatite formation and directly stimulate VICs via phosphate transporters (e.g., Phosphate Transporter 1 (PiT-1)), inducing osteogenic transcription factors such as RUNX2 and BMP2 and leading to matrix mineralization [4,23,29,30]. Clinical cohorts show associations between higher serum phosphate (and in some studies PTH) with accelerated AS progression, particularly in patients with chronic kidney disease with an impaired mineral metabolism [28,29,30]. Additionally, in CKD, hypocalcemia and high PTH levels (enabling uremic cardiomyopathy) were associated with a higher risk of sudden cardiac death [31].

Valvular interstitial cells (VICs) are essential for the structural integrity of the aortic valve. Under pathological stress—mechanical, oxidative, or inflammatory—these cells transdifferentiate into osteoblast-like phenotypes, upregulating osteogenic markers such as bone morphogenetic protein-2 (BMP-2), runt-related transcription factor 2 (RUNX2), and alkaline phosphatase (ALP). This process mirrors vascular calcification and shares similar signals with atherosclerosis [4,5,24]. Under stress, VICs adopt an osteoblastic phenotype, expressing BMP-2, RUNX2, and alkaline phosphatase [23]. Pro-inflammatory cytokines such as IL-6 and TNF-α amplify this reprogramming, while mechanical stress, Oxidized Low-Density Lipoproteins (oxLDL), and Toll-Like Receptor 4 (TLR4) signaling further stimulate calcific nodule formation [4,23,24].

A disturbed calcium–phosphate axis accelerates mineral deposition in valvular tissue. Hyperphosphatemia, commonly present in chronic kidney disease, triggers phosphate uptake through sodium-dependent phosphate transporters PiT-1 and PiT-2, promoting hydroxyapatite formation in VICs [28,29,30]. Clinical observational studies link serum phosphate levels to accelerated calcific aortic valve disease progression and increased need for valve replacement [28,29,30]. Phosphate binders, while widely used in CKD, have not yet been adequately studied in the context of AS [28,30].

Vitamin D signaling exerts context-dependent effects: deficiency correlates with endothelial dysfunction, cardiovascular risk, and may exacerbate inflammation, while active vitamin D metabolites can upregulate osteogenic programs in vascular and valvular cells through the vitamin D receptor (VDR) [21,32,33]. Observational data linking low 25(OH)D to AS severity coexist with mechanistic studies showing VDR-mediated promotion of calcific differentiation under certain conditions, making therapeutic targeting complex [21,33,34]. Vitamin D exhibits a dualistic role in AS. At physiological concentrations, 1,25-dihydroxyvitamin D3 (calcitriol) supports calcium absorption and bone integrity. However, supraphysiological levels or impaired vitamin D receptor (VDR) signaling can paradoxically promote vascular and valvular calcification [21,33]. Ongoing trials (e.g., VITAL-Heart) are exploring optimal vitamin D dosing in cardiovascular prevention, though none have yet focused specifically on AS [34]. Randomized trials of vitamin D supplementation have not demonstrated clear prevention of valvular calcification to date [33,34]. Parathyroid hormone (PTH) modulates bone and mineral homeostasis and can indirectly influence valvular calcification by increasing bone resorption and altering circulating phosphate and calcium [28,30]. Elevated PTH levels in secondary hyperparathyroidism are associated with vascular calcification in CKD and have plausible mechanistic links to VIC osteogenic activation, although direct causal data in AS are limited [28]. Therapeutic modulation (e.g., PTH suppression) remains exploratory for valvular disease.

Vitamin K2 is required for γ-carboxylation of matrix Gla protein (MGP), a calcification inhibitor. In its inactive form, due to vitamin K deficiency or warfarin use, MGP fails to bind calcium, leading to ectopic mineralization [14,25,27,28]. Supplementation with menaquinone-7 has shown promise in attenuating this process, though clinical validation in AS remains ongoing [25,27,28].

Matrix Gla protein (MGP) is a vitamin K-dependent inhibitor of vascular and valvular calcification. MGP must be γ-carboxylated by vitamin K2 (menaquinone) to bind calcium effectively and inhibit hydroxyapatite crystal deposition [14,25,27,28]. Warfarin, a vitamin K antagonist, reduces active MGP levels and accelerates calcification [14,27]. The VitaVasK clinical trial demonstrated that vitamin K2 supplementation may slow progression of cardiovascular calcification, supporting its therapeutic role in aortic valve disease [25,27,28]. The ongoing AVATAR-K2 study is specifically evaluating this strategy in aortic valve calcification [25]. Vitamin K deficiency, whether from dietary insufficiency, CKD, or vitamin K antagonists such as warfarin, leads to accumulation of inactive MGP and unrestrained calcification [14,25,27]. Patients on long-term warfarin therapy exhibit accelerated valve and vascular mineralization, raising concern about its use in patients with AF comorbidity [6,35,36]. Although these mechanisms are supported by robust experimental data and observational clinical associations, randomized trials demonstrating a causal impact on AS or clinical outcomes in AS are currently lacking. Oxidized lipids also contribute; oxidized LDL (oxLDL) promotes VIC mineralization by engaging the NF-κB pathway and enhancing oxidative stress, and lipoprotein(a). (Lp(a)) stimulate TLR4-mediated inflammatory cascades and induce osteogenic reprogramming in VICs [4,10,11,24,37,38]. These molecular insights suggest that calcification in AS is not a passive process, but an active, regulated phenomenon with potential for targeted intervention [1,4,24,39]. Lipoprotein(a) (Lp(a)) is a genetically determined variant of LDL that carries oxidized phospholipids (OxPLs)—potent inducers of VIC activation and calcific nodule formation [10,11,37,38,39]. Genome-wide association studies (GWAS) confirm LP(a) variants as causal for AS, independent of traditional LDL cholesterol, which contributes to calcification via oxidized phospholipids and Toll-like receptor (TLR) activation [11,37,38,39,40,41].

Lp(a) has emerged as a key risk factor for AS. Large randomized trials of statins in moderate-to-severe AS (e.g., SEAS, SALTIRE) failed to slow disease progression despite reductions in LDL, suggesting that once calcification is established statin therapy may be ineffective; timing of intervention (early intervention- pre-symptomatic or mild-to-moderate aortic stenosis, before extensive valvular calcification, irreversible atrial fibrosis, or sustained atrial fibrillation have developed) and the predominant role of Lp(a)/oxidized lipids rather than LDL per se may explain these negative results [4,11,27,38,39]. Emerging approaches— Proprotein Convertase Subtilisin/Kexin Type 9 (PCSK9) inhibitors, antisense oligonucleotides and siRNAs targeting Lp(a), and therapies that reduce oxidized phospholipids—offer targeted strategies that could modify AS risk more effectively than traditional statins and are under active investigation [27,38,39,40,42,43,44,45]. Antisense oligonucleotides (e.g., Pelacarsen) targeting LPA mRNA have shown >90% reductions in Lp(a) levels and are in Phase 3 trials [43,45,46]. While Lp(a)-lowering therapies have demonstrated significant reductions in circulating Lp(a) levels, their ability to slow aortic valve calcification or alter the natural history of aortic stenosis remains unproven and is the subject of ongoing outcome trials. The main metabolic pathways involved in calcific aortic stenosis and atrial remodeling, with their molecular mediators and level of evidence, are summarized in Table 2.

## 4. Epigenetic Regulation: Histone Modification and miRNAs

Histone deacetylases (HDACs), especially HDAC4 and HDAC9, suppress osteogenic inhibitors and promote calcification in VICs [9]. MicroRNAs (e.g., miR-30b, miR-204) inhibit osteogenesis, while others (miR-34a) promote it [9]. Epigenetic reprogramming, reversible through HDAC inhibitors or miRNA mimics, may offer therapeutic potential [9].

## 5. Atrial Remodeling, Metabolomics, and Biomarkers in Aortic Stenosis and TAVR

**Figure 1:**
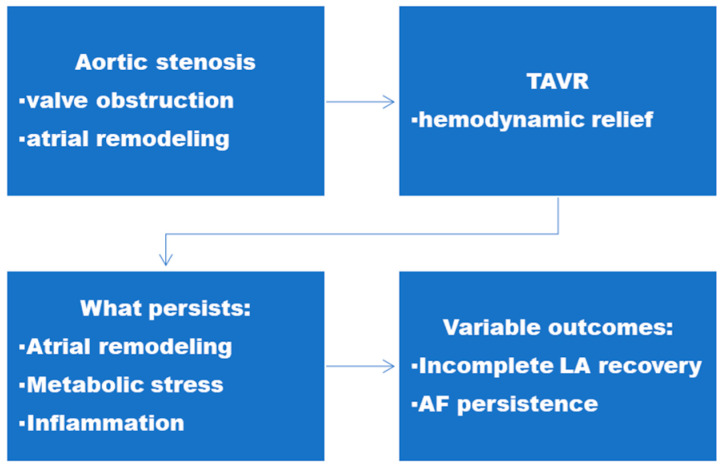
Minimal conceptual model of heterogeneous outcomes after TAVR. LEGEND: Although TAVR relieves valvular obstruction, persistence of atrial remodeling contributes to variable clinical outcomes. ABBREVIATIONS: TAVR—Transcatheter Aortic Valve Replacement; LA—Left Atrium; AF—Atrial Fibrillation.

Hemodynamic overload in AS extends its effects beyond the valve, significantly impacting the atria. Left atrial pressure increases with progressive outflow obstruction, leading to atrial dilation, fibrotic remodeling, and a predisposition to atrial fibrillation (AF) [2,3,6,7]. TGF-β1 and connective tissue growth factor (CTGF) orchestrate fibrogenesis, while galectin-3 and matrix metalloproteinases drive extracellular matrix turnover and collagen deposition [4,15,35]. Chronic left atrial (LA) stretch and elevated atrial wall stress activate mechanosensitive pathways that promote fibroblast-to-myofibroblast differentiation, upregulate transforming growth factor β (TGF β) and other profibrotic cytokines, and drive excess collagen I/III deposition and extracellular matrix remodeling; concomitant neurohormonal activation (RAAS), oxidative stress, and inflammation amplify these processes and create a substrate for conduction heterogeneity and AF [4,7,15,16,17,35]. Persistent atrial fibrosis and metabolic stress may limit atrial reverse remodeling after TAVR, contributing to heterogeneous outcomes, as illustrated in Figure 1.

Mitochondrial dysfunction exacerbates atrial remodeling. Impaired oxidative phosphorylation and fatty acid oxidation result in the accumulation of long-chain acylcarnitines, which impair ion channel function and electrical conduction [16,17,18,19]. Reactive oxygen species (ROS) from dysfunctional mitochondria activate redox-sensitive pathways, reinforcing fibrotic and pro-arrhythmic signaling [4,17,18]. Abnormal fatty acid oxidation and mitochondrial dysfunction result in energy depletion and accumulation of toxic intermediates (e.g., acylcarnitines), promoting atrial fibrosis and arrhythmia [16,17,18]. Long-chain acylcarnitines impair cardiac repolarization and calcium handling [17].

Obesity and Atrial Myopathy: Obesity exacerbates atrial remodeling via RAAS, fatty infiltration, and epicardial adipose tissue–derived cytokines [4,15,35]. Electrical remodeling includes reduced conduction velocity, fibrosis-induced reentry circuits, and AF vulnerability. Fibrosis acts as an electrical insulator, disrupting conduction pathways and favoring reentry circuits [7,35]. LA electrical remodeling is further exacerbated by:

• Downregulation of connexin-40 and connexin-43, reducing gap junction density.
• Altered calcium handling proteins (e.g., RyR2 leak, SERCA2a dysfunction).
• Ion channel remodeling, including downregulation of IK1, Ito, and upregulation of If currents (mixed Na+/K+ inward current, activated by membrane hyperpolarization during early diastole) [7,16,17,35].

Prolonged P-wave duration, PR interval, and electroanatomical low-voltage areas are electrophysiological hallmarks of advanced LA remodeling. These changes increase the likelihood of new-onset AF following TAVR, which occurs in up to 30% of patients and is linked with thromboembolism, heart failure, and death [6,7,15,16,17,46].

Transcatheter aortic valve replacement (TAVR) acutely relieves left ventricular (LV) outflow obstruction and reduces LV wall stress, initiating a cascade of structural, functional, neurohormonal, and metabolic changes; while LV reverse remodeling is well documented- improvements in ejection fraction and diastolic filling, and lower natriuretic peptide levels, left atrial (LA) remodeling—measured as decreased left atrial volume and improved reservoir and conduit strain, follows a distinct and often slower trajectory, and systemic and myocardial metabolic profiles measured by circulating biomarkers and metabolomics—change after valve replacement in ways that may predict or mediate atrial recovery and clinical outcomes [6,7,15,16,21]. But typically to a lesser degree and over a longer time course, with incomplete recovery in patients who have longstanding atrial dilation, significant atrial fibrosis, or persistent atrial fibrillation [6,7,15,16,17].

The metabolic response after TAVR follows an immediate, early and late pattern: acutely there is a fall in filling pressures and natriuretic peptides alongside transient procedural myocardial injury (small rises in troponin), followed over days-to-weeks by reductions in systemic inflammation and neurohormonal activation, and over months by remodeling of myocardial substrate utilization and circulating metabolite profiles; targeted metabolomics studies have documented post-TAVR shifts in amino acid metabolism (including branched-chain amino acids and arginine nitric oxide pathways), reductions in acylcarnitines suggesting improved mitochondrial fatty acid oxidation, and changes in glycerophospholipids and sphingolipids that correlate with functional recovery and symptom improvement [15,16,18,19,20,21].

Recovery of left atrial size and function after TAVR is heterogeneous; strongest predictors of limited atrial reverse remodeling and persistent dysfunction include larger baseline indexed LA volume and worse LA strain, longer duration or presence of atrial fibrillation, imaging evidence of atrial fibrosis (CMR T1/LGE), and elevated biomarkers such as NT proBNP, high sensitivity troponin, and galectin 3—while comorbid conditions (chronic kidney disease, diabetes, pulmonary hypertension), advanced age, and frailty further reduce the likelihood of complete functional recovery [6,7,15,16,17]. Normalization of selected metabolites after TAVR does not uniformly translate into reversal of atrial structural remodeling, particularly in patients with advanced fibrosis or long-standing atrial fibrillation. Observational evidence suggests that elevated circulating acylcarnitines are associated with atrial remodeling and adverse outcomes in patients with AS, suggesting combined imaging biomarker metabolomic models may improve prognostication and patient selection for adjunctive therapies [15,16,18,19,20,21]. To date, Mendelian randomization studies implicate lipoprotein (a) as the strongest genetically supported causal metabolic factor in calcific aortic stenosis. For most other circulating metabolites, including acylcarnitines and amino acids, causal inference remains limited, and associations likely reflect downstream myocardial stress rather than primary drivers.

Metabolomics, using NMR and mass spectrometry, has revealed characteristic metabolic shifts in AS patients. Impaired levels of acylcarnitines, branched-chain amino acids (BCAAs), bile acids, and phospholipids correlate with disease severity and ventricular remodeling [15,16,18,19,20,21]. Post-TAVR, some of these alterations normalize, suggesting potential for dynamic risk monitoring [15,16,19,20,21].

Untargeted metabolomics has identified distinct signatures in AS patients, including elevated acylcarnitines, decreased amino acids, altered phospholipid ratios, and dysregulated bile acids [15,16,18,19,20,21]. These patterns correlate with left ventricular mass index, NT-proBNP levels, and atrial volume [15,16,19,20]. Post-TAVR normalization of select metabolites predicts recovery [15,16,20,21]. Beyond circulating metabolites, imaging-derived and tissue-level metabolic phenotypes also contribute to atrial remodeling. Radiomic and proteomic analyses of epicardial adipose tissue have demonstrated associations with adverse left atrial remodeling and post-interventional atrial fibrillation in AS, supporting a local paracrine metabolic contribution beyond systemic biomarkers [47].

Transcriptomic analyses have identified upregulated osteogenic (e.g., RUNX2, SPP1) and fibrotic (e.g., COL biomarkers) genes in calcified valves, while proteomics have spotlighted osteopontin, periostin, and fibulin-5 as circulating biomarkers [4,20,23]. Epigenomic features such as histone acetylation and microRNA expression (e.g., miR-30b, miR-204) further contribute to disease heterogeneity [9,20].

**Figure 2:**
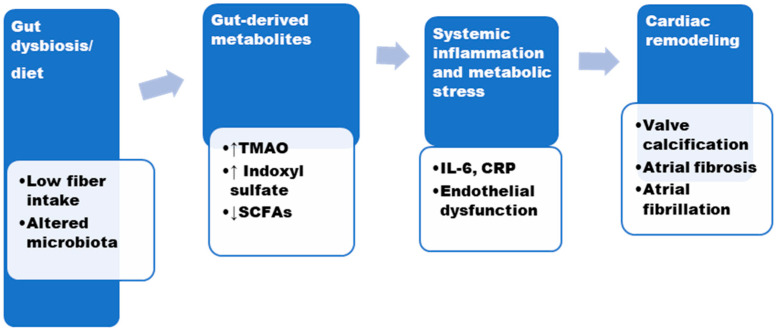
Gut-Heart-Axis in Aortic Stenosis. Legend: Gut microbiota-derived metabolites contribute to systemic inflammation and metabolic dysregulation, promoting valvular calcification and atrial remodeling. Evidence is primarily observational and preclinical. Abbreviations: TMAO—Trimethylamine N-oxide; IL-6—Interleukin-6; CRP—C-Reactive Protein.

Microbial Signatures and Risk Prediction: Elevated TMAO and reduced SCFA-producing bacteria are associated with worse outcomes after TAVR [12,13,25,26]. Multi-omics studies correlate microbial metabolites with atrial volume and fibrosis scores, offering predictive insight for post-procedural complications [15,16,19,20,21]. The gut microbiome is a newly recognized contributor. Trimethylamine-N-oxide (TMAO), derived from microbial metabolism, activates inflammatory and profibrotic cascades via the NLRP3 inflammasome and MAPK pathways [12,13,25,26]. In contrast, short-chain fatty acids (SCFAs) such as butyrate and propionate exert protective effects by regulating immune responses and suppressing histone deacetylase activity [12,25,26]. This emerging gut–heart axis offers new angles for both biomarker discovery and microbiota-targeted therapies [12,25,26]. Conversely, SCFAs like butyrate and acetate exert anti-inflammatory effects via HDAC inhibition and GPR41/43 activation [12,25,26]. Modulating gut microbiota with diet, prebiotics, or microbial enzyme inhibitors (e.g., FMO3 blockers) offers new therapeutic angles [12,25,26]. A conceptual model linking gut-derived metabolites, valve calcification, and atrial fibrosis is presented in Figure 2.

Despite these consistent associations, important interpretative limitations should be acknowledged. Circulating metabolomic profiles likely integrate signals from myocardium, liver, kidney, and skeletal muscle, and do not directly mirror valvular tissue metabolism. Limited studies comparing valve tissue transcriptomics with plasma metabolomics suggest partial overlap but incomplete concordance.

Multi-omics integration, combining genomics, proteomics, metabolomics, and clinical imaging, can identify AS endotypes with differential prognosis [15,16,19,20,21]. Observational evidence suggests that alterations in amino acid metabolism, acylcarnitines, phospholipids, and microbial metabolites are associated with disease severity, atrial remodeling, and outcomes after TAVR. Similar metabolic alterations have been reported in mitral valve disease and heart failure, suggesting that some signatures reflect chronic pressure or volume overload rather than AS-specific biology. Nevertheless, integrating metabolomic data with imaging and clinical phenotyping may help distinguish disease-specific endotypes and improve risk stratification. Most metabolomic signatures identified to date derive from observational cohorts and are influenced by comorbidities, renal function, frailty, and analytical variability, limiting causal inference and immediate clinical translation. Standardization of platforms, validation across independent cohorts, and prospective integration with clinical decision-making remain key unmet needs.

## 6. Glucose Metabolism, Insulin Resistance, and Diabetes

Diabetes mellitus and insulin resistance are associated with a higher prevalence of aortic stenosis and with more rapid progression in observational cohorts [2,3,4]. Hyperglycemia and insulin resistance promote endothelial dysfunction, systemic inflammation, and oxidative stress, which favors VIC activation and calcification [4,23,28,29,30]. Clinical data also link diabetes with worse peri-procedural and mid-term outcomes after TAVR, although risk is influenced by comorbidity burden and frailty [6,7,15,16].

Nonenzymatic glycation of proteins and lipids forms advanced glycation endproducts (AGEs) that cross-link extracellular matrix components, impair matrix turnover, and amplify inflammatory signaling via the receptor for AGE (RAGE); in VICs, AGEs can potentiate osteogenic differentiation and calcific nodule formation [4,23,28,29]. Experimental models show that high glucose or AGE exposure upregulates RUNX2 and other bone-associated genes in valvular cells, while circulating AGEs correlate with valvular calcification in small clinical studies [4,23,28,29]. These mechanisms provide a plausible link between systemic metabolic disease and local valvular mineralization [4,23,28,29,30,33].

## 7. Emerging Therapeutics and Metabolic Modulation

Despite the increasing understanding of aortic stenosis (AS) as an active, metabolic, and inflammatory disease, current treatment remains purely mechanical, surgical, or transcatheter valve replacement [1,2,3,4]. There are no approved pharmacological agents to slow, halt, or reverse the progression of AS or its related atrial remodeling [2,3,4,22]. Targeting mineral metabolism remains an attractive strategy to slow valve calcification, particularly in patients with chronic kidney disease, where phosphate retention, elevated FGF23, and secondary hyperparathyroidism accelerate mineral deposition [28,29,30]; interventions include dietary phosphate restriction, phosphate binders, correction of vitamin D deficiency, and agents that modulate PTH (e.g., calcimimetics) [28,29,30,33]. However, clinical evidence that these measures alter the trajectory of aortic valve calcification or improve clinical outcomes in AS is limited and largely extrapolated from vascular calcification studies in CKD, so routine use for valve disease prevention remains investigational and should be individualized [28,30,33]. Metabolomic profiling could be integrated at multiple points in the clinical pathway for aortic stenosis and TAVR—most promisingly for pre-procedural risk stratification to identify patients at higher risk of incomplete atrial reverse remodeling or adverse events, for peri-procedural monitoring of metabolic perturbations that may reflect myocardial injury or inflammation, and for longitudinal post TAVR surveillance to detect patients who might benefit from adjunctive metabolic or anti-fibrotic therapies [15,16,19,20,21]. Early studies combining metabolomic panels with imaging and conventional biomarkers report improved discrimination for outcomes and atrial recovery, suggesting a role for multimodal risk models in patient selection and personalized follow-up strategies [15,16,19,20,21].

Given the role of lipids and inflammation in early aortic valve disease, lipid-lowering therapies (statins, PCSK9 inhibitors, and emerging Lp(a)-lowering agents) and anti-inflammatory approaches (colchicine, IL-1/IL-6 pathway inhibitors) have been explored as potential disease-modifying treatments [4,11,27,38,39,43,44,45]. Randomized trials of statins in established AS were neutral, likely because intervention occurred late in the disease process; however, recent genetic and mechanistic data implicating Lp(a) and oxidized phospholipids suggest that earlier or targeted lipid-lowering—particularly therapies that reduce Lp(a)—may be more effective at slowing calcific progression [4,11,37,38,39,40,41,45]. Anti-inflammatory strategies show promise in atherosclerotic disease and are being evaluated for valvular indications, but evidence specific to AS and post-TAVR atrial remodeling is currently limited and requires targeted trials [4,15,19,22,39]. Recent experimental and observational data suggest that direct anticoagulants (DOACs), particularly rivaroxaban and dabigatran, may modulate inflammatory pathways implicated in valvular calcification. Duration-dependent reductions in inflammatory markers and calcific activity have been reported in patients with AS receiving rivaroxaban, supported by complementary in vitro findings. Nevertheless, these observations remain hypothesis-generating, and no randomized clinical trials have demonstrated a disease-modifying effect of DOACs on aortic valve calcification or atrial remodeling [48]. Targeting mineral metabolism remains an attractive strategy to slow valve calcification, particularly in patients with chronic kidney disease, where phosphate retention, elevated FGF23, and secondary hyperparathyroidism accelerate mineral deposition [28,29,30]; interventions include dietary phosphate restriction, phosphate binders, correction of vitamin D deficiency, and agents that modulate PTH (e.g., calcimimetics) [28,29,30,33]. However, clinical evidence that these measures alter the trajectory of aortic valve calcification or improve clinical outcomes in AS is limited and largely extrapolated from vascular calcification studies in CKD, so routine use for valve disease prevention remains investigational and should be individualized [28,30,33].

However, emerging therapeutics rooted in metabolic modulation and molecular targeting offer a promising shift in this paradigm [4,9,19,22].

Nutrient modulation holds clinical promise. Omega-3 interventions supported by experimental or early clinical evidence (e.g., Vitamin K2) and speculative or preclinical strategies (e.g., omega-3 fatty acids, magnesium) have all shown efficacy in experimental models for reducing calcification and improving mitochondrial function [18,20,28,29,30,33,36,49,50]. Ongoing trials like AVATAR-K2 and VIKING are evaluating their translational potential [27,36,49].

RNA-based therapies are gaining ground. Antisense oligonucleotides targeting Lp(a) have shown >90% reductions in Lp(a) levels, directly addressing a key calcification driver [40,42,43,44,45]. Preclinical studies have demonstrated that silencing osteogenic signaling pathways, including BMP2, using RNA-based approaches can attenuate calcific response in vascular and valvular cell models [4,9,23].

Senescent VICs contribute to calcification via the senescence-associated secretory phenotype (SASP)—a pro-inflammatory milieu rich in IL-6, IL-8, and matrix metalloproteinases [4,9]. Senescence is triggered by oxidative stress, DNA damage, and telomere attrition, and perpetuates local inflammation and matrix degradation [4,9].

Preclinical data indicate that senolytic agents like dasatinib, quercetin, and navitoclax can selectively eliminate senescent cells and have demonstrated reversal of calcific lesions in experimental models [9]. Senolytic agents, including dasatinib and quercetin, selectively clear senescent cells that drive fibrosis and inflammation. These therapies are currently in phase I/II trials for heart failure and may benefit AS populations by addressing non-reversible remodeling [9,16,19]. Anti-fibrotic agents such as galectin-3 inhibitors and pirfenidone are also under investigation for post-TAVR atrial fibrosis [15,16,19]. To date, the potential role of senolytic or anti-fibrotic therapies in aortic stenosis remains hypothetical, with no clinical trials specifically addressing valvular calcification or post-TAVR atrial remodeling in this population.

### 7.1. Nutrient-Based Interventions: Vitamin K, Magnesium, and Omega-3 Fatty Acids

#### 7.1.1. Vitamin K2 (Menaquinone)

As previously discussed, vitamin K2 activates matrix Gla protein (MGP), a potent inhibitor of calcification. Supplementation aims to restore carboxylated MGP activity, especially in populations with deficiency or warfarin use [14,36,49].

• The AVATAR-K2 trial (NCT04193816) is currently assessing the effect of vitamin K2 supplementation on aortic valve calcification progression [49].
• Dosage and formulation (e.g., MK-7 vs. MK-4) matter due to differing half-lives and tissue penetration [36,50].

#### 7.1.2. Magnesium

Magnesium plays a role in preventing calcium phosphate crystal formation and reduces vascular smooth muscle cell (VSMC) calcification. Oral supplementation has been shown to inhibit hydroxyapatite deposition, possibly via TRPM7 channels and AMPK activation [28,29,30]. Trials in vascular calcification show promise but have not yet been extended to AS specifically [28,30].

#### 7.1.3. Omega-3 Polyunsaturated Fatty Acids (PUFAs)

PUFAs such as eicosapentaenoic acid (EPA) have anti-inflammatory and anti-oxidative effects and improve mitochondrial efficiency [18,20,33]. They reduce TLR4 activation by oxLDL and lower systemic inflammatory markers (CRP, IL-6) [4,24,33]. EPA has been shown to improve left atrial strain and reverse mitochondrial dysfunction in murine models [16,17,18]. Observational evidence suggests an association between these metabolic alterations and disease severity, while preclinical data indicate plausible mechanistic links; however, clinical validation is currently lacking.

### 7.2. Probiotics and Prebiotics

Targeted supplementation with SCFA-producing bacteria (e.g., Bacteroides, Roseburia) may restore gut–heart homeostasis [12,25,26]. Inulin and resistant starch have been shown to increase butyrate levels and reduce IL-6, TNF-α [12,25,26]. Pilot studies are evaluating these interventions in patients with high atrial fibrosis scores pre-TAVR [16,19].

### 7.3. Fecal Microbiota Transplantation (FMT)

FMT remains a highly experimental concept in cardiovascular disease and is currently limited in clinical practice to recurrent, treatment-resistant Clostridium difficile infection. Though still experimental in cardiovascular disease, FMT from healthy donors has reversed hypertension and endothelial dysfunction in mouse models [25,26]. Precision FMT—targeting specific metabolic functions rather than entire microbiota—may provide an avenue for AS-specific interventions in the future [12,25,26]. Despite compelling mechanistic data, evidence linking microbiome-targeted interventions to meaningful clinical outcomes in AS is currently limited to preclinical and early-phase studies.

## 8. Precision Cardiology and Future Directions

Precision cardiology integrates molecular profiling, imaging, and digital health data to personalize AS management [2,3,4,19,22]. For example, omics-guided patient selection for TAVR could optimize timing and minimize complications [15,16,19,20,21,22].

Microbiome-directed interventions such as prebiotics, probiotics, or microbial enzyme inhibitors could reshape systemic inflammation and fibrosis risk [12,13,25,26]. Further studies are needed to define responder phenotypes and optimize timing and dosing strategies [12,25,26].

Patient Stratification via Multi-Omics Integration: Metabolomic profiling could be integrated at multiple points in the clinical pathway for aortic stenosis and TAVR—most promisingly for pre-procedural risk stratification to identify patients at higher risk of incomplete atrial reverse remodeling or adverse events, for peri-procedural monitoring of metabolic perturbations that may reflect myocardial injury or inflammation, and for longitudinal post-TAVR surveillance to detect patients who might benefit from adjunctive metabolic or anti-fibrotic therapies [15,16,19,20,21]. Early studies combining metabolomic panels with imaging and conventional biomarkers report improved discrimination for outcomes and atrial recovery, suggesting a role for multimodal risk models in patient selection and personalized follow-up strategies [15,16,19,20,21].

Multi-omics integration allows clinicians and researchers to go beyond phenotypes to define molecular endotypes of AS [9,19,20,21,22]:

• Calcific-dominant endotypes: High Lp(a), oxLDL, BMP2, and RUNX2 signatures; likely responders to RNA-based or statin therapy [4,11,27,39,40,41,43,45].
• Fibrotic–metabolic endotypes: Elevated TMAO, BCAA, galectin-3; may benefit from anti-fibrotic agents or senolytics [12,13,15,16,19,25,26].
• Inflammatory-senescence endotypes: High IL-6, miR-34a, SASP expression; potential candidates for JAK inhibitors and quercetin [4,9,19,22].

Ultimately, the goal is to delay or prevent TAVR/SAVR in selected patients through mechanism-based therapies [2,3,4,19,22,51]. This will likely involve:

• Early screening with omics and imaging [19,20,22].
• Risk stratification [15,16,19,20].
• Multi-pronged therapies combining nutrient modulation, RNA-based gene silencing, senescence control, and microbiome regulation are conceptual and hypothetical, with no current clinical evidence supporting their combined use in AS [9,12,19,22,25,26,42,44]. Although formal clustering studies remain limited, early multi-omics analyses support the existence of distinct metabolic endotypes with differing trajectories.

Prospective trials such as the VIKING, AVATAR-K2, and LPA-HORIZON studies will help validate these strategies in AS populations [19,27,45,49]. To clarify the distinction between established knowledge, evidence-supported hypotheses, and unresolved uncertainties, the key concepts discussed in this review are summarized in Table 3.

## 9. Limitations

Several limitations should be acknowledged related to the information about metabolic drivers of valve calcification and atrial remodeling in calcific aortic stenosis. First, most of the evidence linking metabolic pathways to valvular calcification and atrial remodeling in aortic stenosis is derived from experimental studies and observational clinical cohorts, which limits causal inference. Many metabolomic and biomarker associations likely reflect downstream myocardial stress, systemic inflammation, or comorbid conditions rather than primary disease drivers. Second, circulating metabolomic profiles integrate signals from multiple organs, including myocardium, liver, kidney, and skeletal muscle, and may not accurately reflect valvular or atrial tissue-specific metabolism. Third, heterogeneity in metabolomic platforms, analytical pipeline, and patient populations limits cross-study comparability and clinical generalizability. Common cardiovascular medications, renal dysfunction, frailty, and dietary factors represent important confounders that may influence metabolic signatures and were not uniformly accounted for across studies. Finally, although emerging therapeutic strategies targeting metabolic, inflammatory, and microbiome-related pathways are biologically plausible, most remain investigational, and no pharmacological intervention has yet demonstrated a disease-modifying effect on aortic valve calcification or post-TAVR atrial remodeling. These limitations underscore the need for standardized, prospective, multi-omics studies and randomized trials to clarify causality and clinical applicability.

## 10. Conclusions

Calcific aortic stenosis is increasingly recognized as a complex, multisystem disease in which valvular calcification, myocardial remodeling, and atrial pathology are interconnected through inflammatory, metabolic, and fibrotic pathways. Beyond progressive obstruction of the left ventricular outflow tract, AS induces maladaptive remodeling of the left atrium, which plays a central role in the development of atrial fibrillation, functional limitation, and adverse outcomes after TAVR.

Although surgical and transcatheter valve replacement remain the only effective treatments for advanced disease, mechanical correction alone does not uniformly reverse downstream atrial remodeling. Persistent atrial dysfunction after TAVR highlights the contribution of pre−existing molecular and metabolic alterations that are not addressed by valve intervention. Accumulating experimental and observational evidence suggests that disturbances in mineral metabolism, lipid oxidation, mitochondrial function, insulin resistance, and gut microbiome-derived metabolites contribute to both valvular calcification and atrial remodeling. However, most available data are associative, and direct causal links and therapeutic implications remain to be established.

Advances in metabolomics and integrated multi−omics approaches have provided valuable insights into the biological heterogeneity of aortic stenosis, revealing metabolic signatures associated with disease severity, atrial remodeling, and recovery after TAVR. At present, these tools should be viewed primarily as instruments for mechanistic understanding and risk stratification rather than as guides for therapeutic decision making. Standardization of analytical platforms, validation across independent cohorts, and prospective evaluation in longitudinal studies are essential before clinical implementation can be considered.

In summary, aortic stenosis should be understood not solely as a valvular disorder, but as a disease involving coordinated metabolic and structural remodeling of the valve and atria. Future progress will depend on integrating molecular profiling with imaging and clinical phenotyping to better define disease endotypes and identify patients at risk of incomplete recovery after valve replacement. Carefully designed prospective studies and randomized trials will be required to determine if targeting metabolic pathways can meaningfully modify disease progression or improve outcomes beyond mechanical valve intervention.

## Figures and Tables

**Table 1:** Conceptual reframing of calcific aortic stenosis. Legend: Conceptual shift from a valve-centered to a metabolic integrative view of calcific aortic stenosis.

Perspective	Traditional View	Active Metabolic-Inflammatory Disease
Disease nature	Passive valvular degeneration	Active, multisystem remodeling
Primary target	Aortic valve	Valve-atrium metabolic axis
Treatment effect	Mechanical obstruction relief	Incomplete reversal of downstream remodeling
Clinical implication	Valve replacement is sufficient	Residual risk beyond valve intervention

**Table 2:** Metabolic and Inflammatory Drivers of Valvular Calcification. Legend: Key metabolic pathways involved in calcific aortic stenosis and their effects on aortic valve calcification and left atrial remodeling.

Metabolic Pathway	Key Molecular Mediators	Effects on the Aortic Valve	Effects on the Left Atrium	Level of Evidence	Clinical Trial/Interventions	Phase Status
Mineral metabolism	Calcium, phosphate, pyrophosphate, matrix Gla Protein, fetuin-A	Osteogenic differentiation of valvular interstitial cells, extracellular matrix remodeling, leaflet calcification	Increased atrial stiffness, interstitial fibrosis	Experimental +observational	Vitamin K2 (menaquinone-7, AVATAR K2)	Phase II-ongoing
Lipid metabolism	Lipoprotein (a), oxidized phospholipids, autotaxin	Inflammatory signaling, VIC activation, and promotion of calcification	Low-grade inflammation, profibrotic signaling	Genetic+observational	Pelacarsen, Olpasiran, Lp(a)-lowering-RNA based therapies	Phase III-ongoing
Mitochondrial dysfunction	Reactive oxygen species, impaired fatty acid oxidation, acylcarnitines	Enhanced oxidative stress, VIC phenotypic switching	Electrical remodeling, reduced conduction reserve, atrial fibrillation substrate	Observational	Metabolic modulation (omega-3 fatty acids; lifestyle interventions)	Preclinical/early phase
Insulin resistance and glucose metabolism	Advanced glycation end-products, branched-chain amino acids, and impaired insulin signaling	Matrix remodeling, accelerated calcification	Structural remodeling, atrial dilation, and increased arrhythmogenic vulnerability	Observational	Glycemic control strategies (indirect evidence)	Observational
Inflammation and innate immunity	Toll-like receptors, NF-Kb signaling, cytokines (IL-6, TNF-α)	VIC activation, promotion of osteogenic pathways	Fibrosis, electrical heterogeneity	Experimental + observational	Colchicine; Colchicine; IL-1β/IL-6 pathway inhibitors	Phase II-hypothesis generating
Gut microbiome derived metabolites	Trimethylamine-N-oxide (TMAO), short-chain fatty acids	Pro-inflammatory and pro-calcific signaling (indirect)	Profibrotic remodeling, systemic inflammation	Preclinic + observational	Dietary modulation, Prebiotics/probiotics	Preclinical/early phase
Cellular senescense	Senescence-associated secretory phenotype, p16, p21	Enhanced calcific signaling, matrix degradation	Fibrotic remodeling, impaired reverse remodeling	Preclinical	Senolytics (Dasatinib, quercetin-experimental)	Preclinical

**Table 3:** From evidence to interpretation: what is known, suggested, and unknown in metabolic aortic stenosis. Legend: Summary of established concepts, evidence-supported hypotheses, and unresolved knowledge gaps relevant to metabolic mechanisms in aortic stenosis and atrial remodeling. Abbreviations: AS: aortic stenosis, Lp(a): lipoprotein a.

Domain	Established	Suggested by Current Evidence	Key Knowledge Gaps
Valvular calcification	Active, cell-mediated process	Metabolic dysregulation contributes	Modifiability without valve replacement
Atrial remodeling	Predicts atrial fibrillation and outcomes	Partially independent of valve obstruction	Reversibility after early intervention
Metabolic alterations	Associated with disease severity	Reflect systemic myocardial stress	Causality vs. epiphenomenon
Metabolomic	Enables phenotyping	Risk stratification potential	Clinical validation, standardization
Targeted therapies	Valve replacement effective	Lp(a) lowering biologically plausible	Impact on AS progression

## Data Availability

Not applicable.

## Abbreviations

The following abbreviations are used in this manuscript:

AF	Atrial Fibrillation
AGEs	Advanced Glycation End Products
ALP	Alkaline Phosphatase
AMPK	AMP-Activated Protein Kinase
AS	Aortic Stenosis
BCAAs	Branched-Chain Amino Acids
BMP2	Bone Morphogenetic Protein-2
CKD	Chronic Kidney Disease
CMR T1/LGE	Cardiac Magnetic Resonance T1 mapping/Late Gadolinium Enhancement
COL1A1	Collagen Type I Alpha 1 Chain
CRP	C-Reactive Protein
CTGF	Connective Tissue Growth Factor
DNA	Deoxyribonucleic Acid
EPA	Eicosapentaenoic Acid
FMT	Fecal Microbiota Transplantation
FMO3	Flavin-Containing Monooxygenase 3
GWAS	Genome-Wide Association Studies
HDACs	Histone Deacetylases
IL-6, IL-8	Interleukin-6, Interleukin-8
IK1, Ito, If	Cardiac Ion Currents
LA	Left Atrium
Lp(a)	Lipoprotein(a)
LV	Left Ventricle
MAPK	Mitogen-Activated Protein Kinase
MGP	Matrix Gla Protein
miRNA/miR	MicroRNA
NLRP3	NOD-Like Receptor Pyrin Domain–Containing Protein 3
NMR	Nuclear Magnetic Resonance
NT-proBNP	N-Terminal pro-B-Type Natriuretic Peptide
oxLDL	Oxidized Low-Density Lipoprotein
OxPLs	Oxidized Phospholipids
PCSK9	Proprotein Convertase Subtilisin/Kexin Type 9
PiT-1/PiT-2	Phosphate Transporter 1/2
POSTN	Periostin
PUFAs	Polyunsaturated Fatty Acids
PTH	Parathyroid Hormone
RAAS	Renin–Angiotensin–Aldosterone System
RAGE	Receptor for Advanced Glycation End Products
RNA	Ribonucleic Acid
ROS	Reactive Oxygen Species
RUNX2	Runt-Related Transcription Factor 2
RyR2	Ryanodine Receptor 2
SASP	Senescence-Associated Secretory Phenotype
SCFAs	Short-Chain Fatty Acids
SERCA2a	Sarcoplasmic Reticulum Ca^2+^-ATPase 2a
siRNA	Small Interfering RNA
SPP1	Secreted Phosphoprotein 1 (Osteopontin)
TAVR	Transcatheter Aortic Valve Replacement
TGF-β1	Transforming Growth Factor Beta 1
TMAO	Trimethylamine N-oxide
TLR4	Toll-Like Receptor 4
TRPM7	Transient Receptor Potential Melastatin 7
VDR	Vitamin D Receptor
VICs	Valvular Interstitial Cells
VSMC	Vascular Smooth Muscle Cell
